# Antibiotic Resistance and Molecular Characterization of *Staphylococcus aureus* Strains Colonizing the Nose and Pharynx

**DOI:** 10.3390/microorganisms13091978

**Published:** 2025-08-25

**Authors:** Samuel González-García, Anaíd Bustos-Hamdan, Aída Hamdan-Partida, Jaime Bustos-Martínez

**Affiliations:** 1 Doctorado en Ciencias Biológicas y de la Salud, Universidad Autónoma Metropolitana, Mexico City 04960, Mexico; sgonzalezg@correo.xoc.uam.mx; 2 Departamento de Atención a la Salud, Universidad Autónoma Metropolitana-Xochimilco, Mexico City 04960, Mexico; abustos@correo.xoc.uam.mx (A.B.-H.); ahamp@correo.xoc.uam.mx (A.H.-P.)

**Keywords:** *Staphylococcus aureus*, colonization, nose, pharynx, virulence genes, antibiotic resistance, *spa*-typing, SCC*mec*

## Abstract

*Staphylococcus aureus* colonizes the nasal and pharyngeal mucosa of a considerable proportion of the human population, representing a relevant reservoir for the dissemination of antibiotic resistance. This study aimed to determine the prevalence, antibiotic resistance profiles, and molecular characterization of *S. aureus* strains isolated from the nose and pharynx of healthy individuals of Mexico City, Mexico. Nasal and pharyngeal swabs were taken from 1777 individuals aged between 1 and 99 years. Microbiological identification, antibiotic susceptibility testing, virulence gene detection, SCC*mec* typing, and *spa*-typing allowed the characterization of 1459 strains. *S. aureus* colonization was found in 59.7% of the population, with more of these strains being found in the pharynx than in the nose. MRSA constituted 9.25% of the strains, with a predominance of strains with SCC*mec* types IV and IVa. The most frequent resistance of *S. aureus* strains was to penicillin (87.6%), clindamycin (43.4%) and erythromycin (27.2%), with no statistical differences regarding the anatomical sites of isolation. The most frequently found virulence genes were *lukE-D*, *icaA*, *sdrC*, *coa*, and *gyrA*. Sixty-two *spa*-types were identified, and t-189 was the most common. These findings reflect a high colonization rate and genetic diversity, highlighting the importance of considering both anatomical sites in *S. aureus* surveillance studies.

## 1. Introduction

*Staphylococcus aureus* is a Gram-positive bacterium that colonizes about half of the human population persistently or intermittently as a commensal bacterium without presenting symptoms [1,2]. However, this microorganism can trigger clinical problems with variable pathology, ranging from mild to moderate infections such as endocarditis, osteomyelitis, infectious arthritis, abscesses, and biofilm infections on medical devices, to life-threatening infections such as sepsis [3]. The molecular basis for the transition of *S. aureus* strains from commensal to pathogenic is still under investigation [1]. One of the main problems of *S. aureus* is antibiotic resistance, especially methicillin-resistant strains (MRSA), both community-acquired (CA-MRSA) and hospital-acquired (HA-MRSA). These strains generally also exhibit resistance to several classes of antimicrobials [4].

The most studied niche of *S. aureus* in humans is the nose, with reported incidences of 20–80% [5], but it also colonizes other anatomical sites in varying percentages, such as the intestines (17–31%), vagina (22%), perineum (22%), abdomen (15%), and axillae (8%) [6,7,8]. However, another important site is the pharynx, which presents variability in the colonization percentage with reports from 4% to 75% [9], and pharyngeal colonization may even be higher than nasal colonization in studies where nasal and pharyngeal samples are taken at the same time [5,6,10,11,12,13].

This study was conducted in a Mexican population. In Mexico, there are few studies on the molecular characteristics and antibiotic resistance of *S. aureus* strains isolated from the pharynx and nose. This research aims to describe the incidence of *S. aureus*, as well as its antibiotic resistance, and the molecular characterization of MRSA isolated from the pharynx and nose.

## 2. Materials and Methods

### 2.1. Sampling and Identification of S. aureus

Pharynx and nasal swabs were collected from 1777 apparently healthy male and female participants, aged 1 to 99 years, in daycare centers, schools, industries, and nursing homes in Mexico City, between 2018 and 2023. All participants provided their informed consent to participate as volunteers; in the case of minors, their parents signed the informed consent. No incentives were offered. The project was approved by the Ethics Committee of the Biological Sciences and Health Division of the UAM-Xochimilco (Document: DCBS.CD.056.18).

The swabs were incubated in tubes with trypticase soy broth (BD Bioxon, Mexico City, Mexico) at 37 °C for 24 h and then plated on mannitol salt agar (BD Bioxon, Mexico City, Mexico) using the cross-streak method to isolate and microbiologically identify mannitol-fermenting colonies. Strains were identified as *S. aureus* when they were positive to the mannitol fermentation test and the coagulase test [9,12]. This identification was completed by sequencing the 16S rRNA gene following the methodology described [14]. The amplicons were sent to Macrogen Korea for sequencing and aligned themselves in the BLAST program (https://blast.ncbi.nlm.nih.gov/Blast.cgi (accessed on 18 October 2023).

### 2.2. Identification of Antibiotic-Resistant Strains and MRSA

All strains identified as *S. aureus* were subjected to the disk diffusion test for antibiotics against Gram-positive bacteria using a polydisc (PBM, Mexico City, Mexico) with ciprofloxacin (CIP, 5 μg), fosfomycin (FO, 50 μg), trimethoprim-sulfamethoxazole (TSX, 25 μg), penicillin (P, 10 μg), vancomycin (VA, 30 μg), tetracycline (TE, 30 μg), erythromycin (E, 15 μg), oxacillin (OX, 1 μg), nitrofurans (MAC 300 μg), clindamycin (CC, 2 μg), gentamicin (GM, 15 μg), and cephalothin (CF, 30 μg). The procedure was performed in Muller-Hinton medium (BD Bioxon, Mexico City, Mexico) at a concentration of 0.5 of the McFarland scale (approximately 1.5 × 10^8^ CFU/mL), using a densitometer (Densimat, Biomèriux, Craponne, France); the dishes were incubated at 37 °C for 24 h. Strains were classified as susceptible, resistant or intermediately resistant. *S. aureus* strains ATCC 43,300 and ATCC 29,213 were used as controls. The procedure and interpretation of results followed the guidelines of the Clinical Laboratory Standards Institute (CLSI, 2018) [15].

To identify MRSA, the oxacillin Minimum Inhibitory Concentration (MIC) test was performed following the CLSI methodology [16]. Strains that grew at a concentration ≥ 4 µg/mL of oxacillin were considered MRSA, *S. aureus* strain ATCC 43,300 was used as a positive control, and ATCC 29,213 was used as a negative control. In addition, the presence of the *mecA* gene was determined by PCR following the methodology reported by Oliveira et al. [17] for all strains that tested positive in the oxacillin MIC test.

### 2.3. Virulence Gene Typing of MRSA

MRSA were identified by the type of SCC*mec* they presented by multiplex PCR according to the methodology of Oliveira et al. [17] and Boye et al. [18], using as controls the *S. aureus* strains BAA-44, BAA-41, BAA-39, NRS 643 and NRS 745 as positive controls for SCC*mec* types I, II, III, IV, and V, respectively [19]. Strains that were typed as SCC*mec* types IV, Iva, and V and present the Panton-Valentine leucocidin (PVL) gene were classified as CA-MRSA as mentioned in the literature [19,20,21].

The following virulence genes were detected by PCR. The genes of the enzymes: the *arcA* gene, which is located in the arginine catabolic mobile element (ACME) and encodes arginine deiminase [22]; the thermonuclease gene (*nucA*) [23]; the *gyrA* gene that encodes the A subunit of DNA gyrase [24]; the coagulase protein gene (*coa*) [25]. The adhesin genes: fibronectin-binding *fnbA* [26], and *fnbB* [27]; the collagen-binding protein gene (*cna*) [26]; the aggregation factor A and B genes (*clfA* and *clfB*) [26]. Genes responsible for biofilm formation: *icaA* and *icaD* genes of the *icaADBC* operon [26,28], the serine-aspartate repeat protein C gene (*sdrC*) [29], and the phenol-soluble modulin gene (*psm*) [30]. The enterotoxins A, B, C, D, and E genes (*sea*, *seb*, *sec*, *sed*, and *see*) [31], exfoliative toxins (*eta* and *etb*) [31], the hemolysins genes (*hla*, *hlb*, *hlg*, and *hld*) [31], and the toxic shock syndrome toxin-1 gene (*tst*) [31]. The presence of Panton-Valentine leucocidin (PVL) was determined by amplification of the *lukS-PV/lukF-PV* genes [31]; *lukE-D* [31] and *luk-M* genes were also detected [32]. The primers used for gene amplification are presented in Table S1.

*S. aureus* strains were typed (*spa*-typing) by PCR amplification of the *spa* gene [33], and amplicon sequencing at MacroGen (Seoul, Republic of Korea), *spa*-types were assigned using the SPA Searcher (http://seqtools.com (accessed on 20 November 2024)) and the Ridom GmbH website (http://spaserver.ridom.de/ (accessed on 20 November 2024)).

### 2.4. Statistical Analysis

Descriptive statistics were used for the analysis of quantitative and categorical variables. Pearson’s chi-square test was used to evaluate associations between categorical variables, such as carrier type, anatomical site of isolation, virulence gene distribution, and antimicrobial resistance patterns. Fisher’s exact test was employed in cases where the expected frequencies were less than five. The Z test was used to compare specific proportions between two independent groups. All analyses were performed using SPSS Statistics 25.0 (IBM, Armonk, NY, USA). A *p* < 0.05 was considered statistically significant.

## 3. Results

### 3.1. Sampled Population

Pharyngeal and nasal swabs were taken from 981 women (55.2%) and 796 men (44.8%) (N = 1777), with an average age of 22.49 years. The population was divided into 10 groups by age intervals, with 499 people between 1 and 10 years (28.08%), 557 between 11 and 20 years (31.34%), 329 individuals from 21 to 30 years (18.51%), 165 people between 31 and 40 years (9.28%), 105 people from 41 to 50 years (5.90%), 49 individuals between 51 and 60 years (2.75%), and 73 people between 61 and 99 years (4.12%) (Table 1).

### 3.2. Carriers of S. aureus

**Figure 1:**
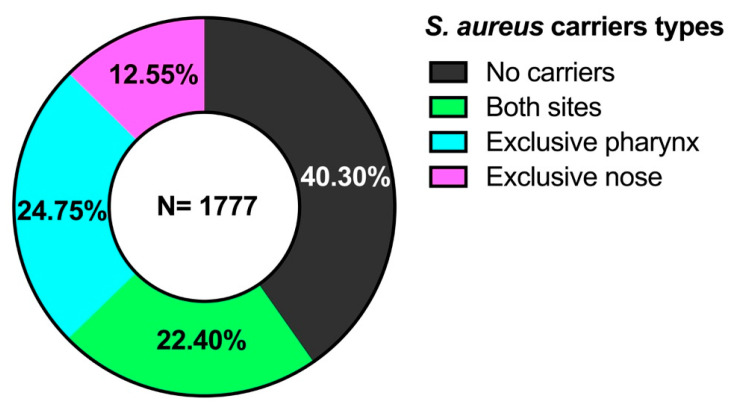
Carriers and non-carriers of *S. aureus* by anatomical site of isolation.

In the study population, 716 non-carriers (40.30%) and 1061 carriers of *S. aureus* (59.70%) were identified (*p* < 0.05). When analyzed by gender, 625 female carriers and 436 male carriers were found (*p* < 0.05). A total of 398 carriers presented the bacteria in both sites, the pharynx, and the nose (22.40%), in 440 individuals *S. aureus* was isolated exclusively in the pharynx (24.75%), while 223 exclusive nasal carriers were found (12.55%) (*p* < 0.05) (Figure 1). A total of 1459 *S. aureus* strains were isolated, of which 838 were isolated from the pharynx (47.43%) and 621 from the nose (42.57%).

### 3.3. Antibiotic Resistance

**Figure 2:**
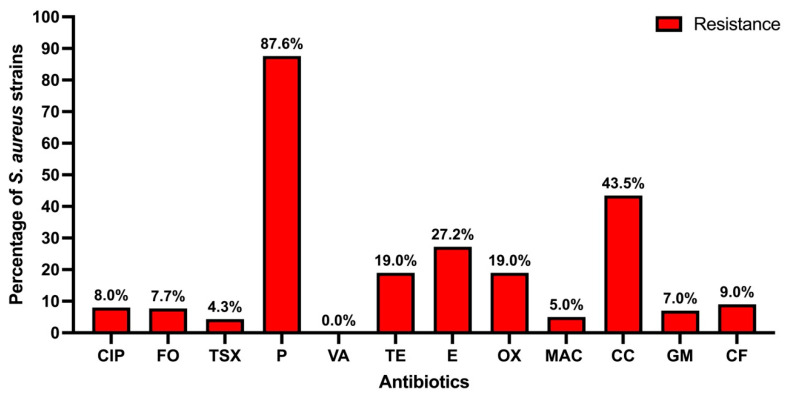
Antibiotic resistance of isolated *S. aureus* strains. Ciprofloxacin (CIP), fosfomycin (FO), trimethoprim-sulfamethoxazole (TSX), penicillin (P), vancomycin (VA), tetracycline (TE), erythromycin (E), oxacillin (OX), nitrofurans (MAC), clindamycin (CC), gentamicin (GM) and cephalothin (CF).

Regarding antibiotic resistance, it was found that 87.60% of the strains are resistant to penicillin, in second place 43.37% of the strains presented resistance to clindamycin, while the third antibiotic with the highest resistance was erythromycin (27.24%). Following that, 19% were resistant to oxacillin and tetracycline, the other antibiotics presented less than 10% of resistant strains, and no strains resistant to vancomycin were found; these results are presented in Figure 2.

**Figure 3:**
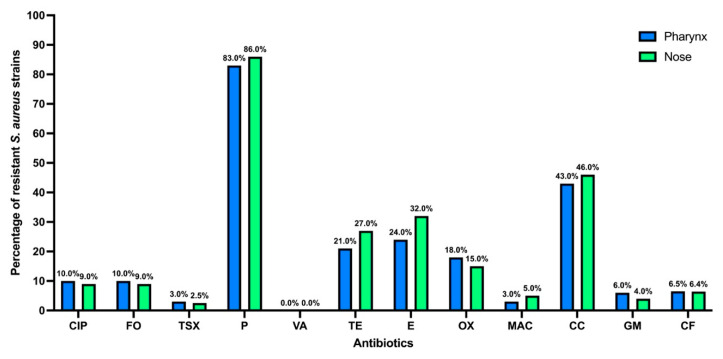
Antibiotic resistance of *S. aureus* strains isolated from the pharynx and nose. Ciprofloxacin (CIP), fosfomycin (FO), trimethoprim-sulfamethoxazole (TSX), penicillin (P), vancomycin (VA), tetracycline (TE), erythromycin (E), oxacillin (OX), nitrofurans (MAC), clindamycin (CC), gentamicin (GM), and cephalothin (CF).

**Figure 4:**
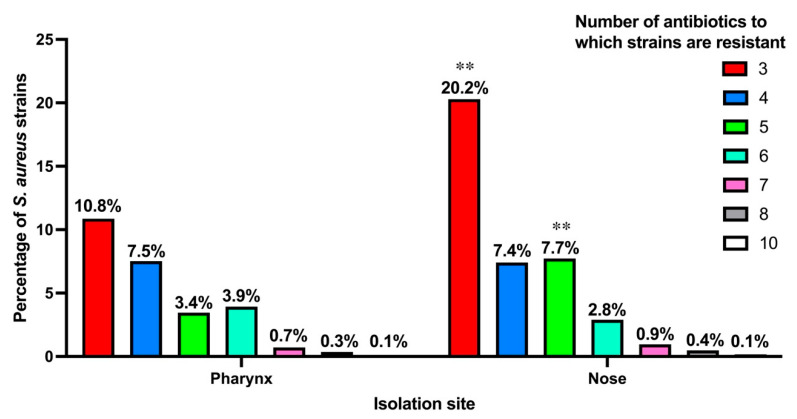
Number of antibiotics to which *S. aureus* strains are resistant. ** *p* < 0.01.

When separating the antibiotic-resistant *S. aureus* strains by anatomical site of isolation, similar results to those in Figure 3 are observed, no statistical differences were found between nasal and pharyngeal strains in any of the antibiotics analyzed (*p* > 0.05) (Figure 4); however, the strains isolated from the nose showed a higher percentage of resistance to penicillin, tetracycline, erythromycin, and clindamycin.

No *S. aureus* strain was found to be sensitive to all antibiotics; all strains showed resistance to at least one antibiotic. Strains resistant to a single antibiotic were found in 33.2% in the pharynx and 25.7% in the nose, while those resistant to two antibiotics were found in 39.8% and 34.3% in the pharynx and nose, respectively. A total of 474 strains (32.48%) presented resistance to three or more antibiotics, of which 248 were nasal strains and 226 were pharyngeal strains (*p* > 0.05); the main combination of resistance was penicillin, erythromycin and clindamycin (115 strains).

Figure 4 shows the percentages of strains that showed resistance to three or more antibiotics in both the pharynx and nose. It is observed that 20.2% of nasal strains showed resistance to three antibiotics, compared to 10.8% of pharyngeal strains (*p* < 0.01). Significant statistical differences are also observed when comparing strains resistant to five antibiotics, where 7.7% of nasal strains are found against 3.4% of pharyngeal strains. (*p* < 0.01). No differences were found between the percentage of strains with resistance to 4, 6, 7, 8, and 10 antibiotics (*p* > 0.05).

### 3.4. Types of S. aureus Carriers by Age Group

**Figure 5:**
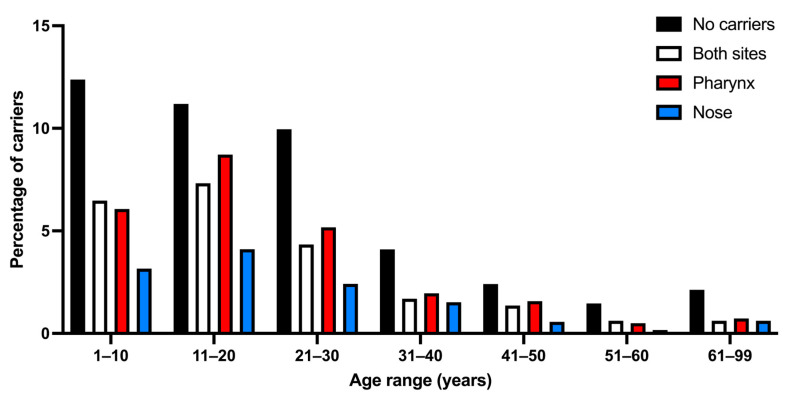
Percentage of carriers and non-carriers of *S. aureus* by age group.

A total of 59.7% of the various types of *S. aureus* carriers were found (Table 1). The percentage of carriers among age groups ranged from 46.94% to 64.44%, decreasing with age. When comparing the percentage of carriers versus non-carriers, only a few age groups showed significant differences (11–20, 21–30, and 41–50 years) (Table 1). Regarding *S. aureus* carriers at both sites, very similar results were found in all groups, with percentages between 15 and 23% (*p* > 0.05). About exclusive nasal and pharyngeal carriers, it can be seen that exclusive pharyngeal carriers are greater (24.75%) than exclusive nasal carriers (12.55%) (*p* < 0.01) (Table 1). These results can be seen in Figure 5.

### 3.5. Identification of MRSA

Of the 1459 isolated *S. aureus* strains, only 135 were typed as MRSA (9.25%), and were isolated from 6.19% of the study population (110 participants), of which 50 strains were isolated from carriers at both sites (3.42%), while 42 strains were found exclusively in the pharynx (2.87%) and 43 strains exclusively in the nose (2.94%). Table 2 shows the grouping of MRSA from participants ordered by age group and isolation site, finding no significant differences between pharynx and nose, or in age groups (*p* > 0.05).

The 135 MRSA presented the *mecA* gene, regarding the type of SCC*mec*, 32 SCC*mec* type II strains were found (23.70%), 1 SCC*mec* type III strain (0.74%), 71 SCC*mec* type IV strains (52.59%), 28 as SCC*mec* type IVa (20.74%) and 3 SCC*mec* type V strains (2.22%), no SCC*mec* type I strain was found, so there were more MRSA with SCC*mec* types IV, IVa or V (75.56%), than MRSA strain with SCCmec type II or III (24.44%) (*p* < 0.0001).

Regarding the isolation site, of the 67 pharyngeal strains isolated, 19 were SCC*mec* type II (28.35%), 36 SCC*mec* type IV (53.73%), 11 SCC*mec* type IVa (16.41%), and only one pharyngeal strain was typed as SCC*mec* type V (1.47%). While in the case of the 68 nasal strains, 13 were SCC*mec* type II (19.11%), one was SCC*mec* type III (1.47%), 35 were SCC*mec* type IV (51.47%), 17 were SCC*mec* type IVa (25%), and only two nasal strains were typed as SCC*mec* type V (2.94%); no significant differences were found when comparing SCC*mec* type and the isolation site (*p* > 0.05) (Table 3).

### 3.6. Molecular Characterization of MRSA

**Figure 6:**
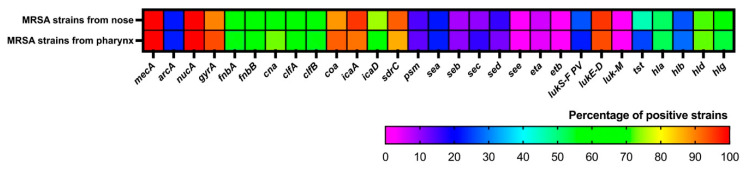
Heat map of the percentage of virulence genes present in the isolated MRSA.

The results of the presence of virulence factor genes amplified by PCR from MRSA isolated from the pharynx and nose are presented in Figure 6 and Supplementary Table S2. It is observed that the most prevalent genes are *mecA*, *lukE-D*, *icaA*, *sdrC*, *coa*, and *gyrA*, which are present in at least 90% of MRSA. The genes *nucA*, *fnbA*, *fnbB*, *cna*, *clfA*, *clfB*, *icaD*, *sdrC*, *gyrA*, *coa*, *hla*, *hld*, *hlg*, and *tst* were found in between 25% and 90% of MRSA. While the genes with a presence of less than 25% are *arcA*, *psm*, *lukS-PV/lukF-PV*, the five enterotoxins (*sea*, *seb*, *sec*, *sed*, *see*), and the two exfoliative toxin genes (*eta* and *etb*). Only the *luk-M* gene is not present in any of the strains, while the *see* gene was amplified in only one pharyngeal strain and was not amplified in nasal strains (Table S2).

In general, toxin genes are found in smaller proportions in the MRSA analyzed, except for the *tst* gene, which presents significant differences between pharyngeal and nasal strains (*p* < 0.05) (Table S2), being found in a higher percentage in nasal strains (Figure 6). Both adhesin and hemolysin genes are present in a medium percentage and do not differ depending on the strain isolation site. Genes involved in biofilm formation (*icaA*, *icaD*, and *sdrC*) are present in a high percentage, and a greater presence is also observed in strains isolated from the nose compared to strains isolated from the pharynx (Figure 6).

On the other hand, only 28 strains were found (12 in the pharynx and 16 in the nose) that presented the characteristics of CA-MRSA, that is, they present both SCC*mec* type IV, Iva, or V and the PVL genes. Therefore, only 20.7% of the MRSA isolated are CA-MRSA.

**Figure 7:**
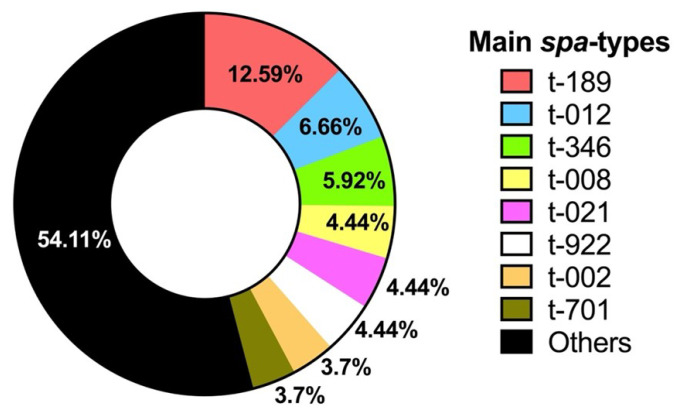
Percentage of MRSA with the main *spa*-types found.

MRSA were also typed using the *spa* gene (*spa*-typing), 62 *spa*-types were found, the most prevalent being t-189 with 17 strains (6 nasal and 11 pharyngeal), followed by t-012 with 9 strains (6 nasal and 3 pharyngeal), t-346 presented 8 strains (2 nasal and 6 pharyngeal), t-922 presented 6 strains (4 nasal and 2 pharyngeal), t-021 and t-008 with 6 strains too (3 nasal and 3 pharyngeal), and the fifth most prevalent type with 5 strains were t-002 (5 pharyngeal) and t-701 (3 nasal and 2 pharyngeal). Figure 7 shows the main *spa*-typing of the isolated MRSA. The complete list of *spa*-types is shown in Supplementary Table S3.

## 4. Discussion

*S. aureus* colonization in the healthy population studied is high, it was found that the colonization of pharyngeal carriers is higher than that of nasal carriers. Similarly, several investigations highlight a higher percentage of colonization in the pharynx than in the nose [5,6,9,10,11,12,13,34,35], which reinforces the fact that the pharynx is an important site of *S. aureus* colonization. It is important to mention that the vast majority of published studies only evaluate nasal colonization of *S. aureus*; however, more and more studies report the importance of the pharynx as an important site that should be taken into account for the detection of this microorganism.

Analysis of data by age group reveals trends in *S. aureus* colonization. In the younger age groups (1–10 years), a higher prevalence is observed when it colonizes both sites and when it colonizes the pharynx exclusively. In adults aged 31–40 and 41–50 years, a reduction in colonization is seen in both sites. This also occurs in older age groups (51–60 years and 61–99 years), where a significant drop in colonization is observed in both anatomical sites.

These trends suggest that with age, the incidence of *S. aureus* carriage decreases, particularly in dual-site and nasal-only carriers, whereas the proportion of non-carriers progressively increases; this is in agreement with that reported by Kuehnert et al. [36], who investigated the nasal prevalence of *S. aureus* in 9622 American population aged 1 to over 60 years, reporting that nasal colonization is most common in participants aged 6–11 years, decreasing progressively with age.

While a meta-analysis based on a systematic literature review of the prevalence and risk factors associated with *S. aureus* colonization in healthy individuals in underdeveloped and developing countries, [37] found that compared with children and younger adults, the elderly consistently showed lower rates of *S. aureus* colonization, especially in community-dwelling populations. This may be because the nasal and pharyngeal microbiome has been observed to change with age and in older adults there is a reduction in microbial diversity [9,38].

Regarding antibiotic resistance, a high percentage of isolated *S. aureus* strains are resistant to penicillin, followed by clindamycin, erythromycin, and tetracycline. Resistance to other antibiotics is less than 10%. These results are similar to those presented by Locke et al. in a meta-analysis performed on healthy individuals [37]. On the other hand, no statistically significant differences were found between antibiotic resistance and the site of isolation of *S. aureus*, suggesting that resistance does not depend on the site of colonization.

It is also important to mention that there is a high percentage of strains resistant to three or more antibiotics, only a significant statistical difference was found with respect to the isolation site between the strains that presented resistance to three and five antibiotics, in both cases the nasal strains were the ones that presented the highest percentage with respect to the pharyngeal ones.

Regarding MDR strains, in this population we found a high number of strains that presented a combination of resistance to penicillin (beta-lactam), erythromycin (macrolide), and clindamycin (lincosamide); in other cases, resistance to ciprofloxacin (fluoroquinolone) was added. Strains with resistance to penicillin (beta-lactam), tetracycline, trimethoprim-sulfamethoxazole (antifolate), and gentamicin (aminoglycoside) were also found. In addition, strains with resistance to several other classes of antibiotics were found, which reinforces the WHO’s concern regarding *S. aureus* as a priority pathogenic bacterium of importance in public health [39].

MRSA are included in the WHO list of high-priority pathogenic bacteria; in our study, a percentage of 9.25% of MRSA was found. The percentage of MRSA in this study is similar to other studies [9,10,40], but differs from other studies where they find lower percentages [6,41,42,43,44], these differences could be mainly due to the age and number of people studied, socioeconomic factors and health measures of each country. Furthermore, no statistically significant differences were found regarding the percentage of MRSA either by age group or by isolation site, implying that these types of strains can be found at any age and colonization site.

Methicillin resistance is associated with the *mecA* gene that encodes the penicillin-binding protein 2a (PBP2a). This gene is located in the staphylococcal cassette chromosomal *mec* (SCC*mec*). It has been found that the main types of SCC*mec* in clinical isolates are types II, IV, and III, while MRSA SCC*mec* type V strains are not frequent [45]. This coincides with the results of this work, where SCC*mec* type IV was the most frequently found type in strains from both niches, followed by SCC*mec* types II and IVa, while SCC*mec* type V was the least prevalent. We also found more pharyngeal strains with SCC*mec* type II compared to nasal strains. It is important to mention that no SCC*mec* type I strain was typed, which indicates little or no circulation of this type in the population analyzed.

In addition, CA-MRSA were typed, they presented both SCC*mec* type IV or IVa as well as the *lukF-PV/lukS-PV* genes. In addition, epidemiological criteria indicate that the individuals should not have been hospitalized [20,21,46]. In this case, individuals who were in schools, factories, daycare centers, or nursing homes were studied; they were not in hospital environments, so the characteristics of CA-MRSA were met. Only 2.6% of carriers presented CA-MRSA, and this value is within the percentage found in a meta-analysis on the prevalence of MRSA [47]. CA-MRSA were found in both the nose and pharynx, so the niche is not a limiting factor for colonization of these types of strains, as has been previously reported [12].

Unlike many other bacterial pathogens, which often rely on only one or a few toxins to promote disease, *S. aureus* produces a wide variety of virulence factors that can threaten human health [1,13,48]. In this work, we investigated the presence of diverse virulence factor genes such as adhesins (*fnbA*, *fnbB*, *cna*, *clfA*, *clfB*, *coa*), toxins (*sea*, *seb*, *sec*, *sed*, *see*, *eta*, *etb*, *hla*, *hlb*, *hld*, *hlg*, *lukS-PV/lukF-PV*, *lukE-D*, *lukM)*, enzymes (*arcA*, *nucA*, *gyrA*), and biofilm formation (*icaA*, *icaD*, *sdrC*, *psm*) in the isolated MRSA.

The enterotoxin genes were found in a low percentage and the exfoliative toxin genes are present in very few strains; however, the toxic shock syndrome toxin-1 gene was found in a higher percentage, and these results are similar to those reported by Kot et al. [49], with the difference that their MRSA were isolated from hospitals and ours are from an apparently healthy population, so it seems that the presence of toxin genes is not altered by the environment. However, we found that there is a significant difference in the *tst* gene and the isolation niche, finding a higher percentage in nasal strains.

*S. aureus* strains that produce the toxic shock syndrome toxin-1 (TSST-1), especially MRSA, are associated with high mortality in bacteremia and sepsis [50]. This toxin aggravates other diseases such as eczema herpeticum, necrotizing pneumonia, and septic arthritis [51,52]. Strains that contain this toxin pose a risk to public health and therefore require surveillance.

The leucocidin genes, especially from PVL, were found in a low percentage, the *luk M* gene was not found in any of the strains studied, while the *luk E-D* gene was found in almost all strains, a result similar to that found in Spanish medical students where the presence of this gene was present in 100% of MRSA isolated from the nose [53], as well as a study in the USA where more than 90% of nasal strains presented the *luk E-D* gene [54].

Leukocidin ED is the most common leukotoxin found in clinical isolates [55,56]. LukED is associated with diabetic foot infections [57], and with the co-expression of other virulence factors such as biofilms in osteomyelitis [58], so its detection is important.

In the case of hemolysin genes, we found a prevalence in descending order of *hld*, *hlg*, *hla*, and *hlb* in MRSA and no differences were found with the isolation site. This is similar to a study in Iran where they report the presence of these genes in MRSA from clinical samples [59]; however, another Iranian study carried out in 2020 on MRSA nasal isolates detected a high presence of the *hla* and *hlb* genes (88.18% and 62.07%, respectively), a higher percentage than the one we found [60], which are differences that could be due to the environment where the strains were isolated.

Adhesin genes were generally found in medium-high percentages (between 57% and 71%), as in other studies that present similar percentages regarding the presence of the *fnbA*, *fnbB*, *clfA*, *clfB*, and *cna* genes [61,62]. Regarding the genes involved in biofilm formation (*icaA*, *icaD*, and *sdrC*), they were found in a high percentage of MRSA, and the percentage is higher in nasal strains compared to pharyngeal strains. Adhesins and biofilm formation are mechanisms that can favor the colonization and persistence of this microorganism [9,63], so their presence should be necessary in most strains as is found in this work.

The distribution of virulence genes of *S. aureus* isolated from human skin and nose has been analyzed by pangenomic studies [64,65], and it has been found that strains share a large repertoire of genes but show different phenotypes, suggesting that *S. aureus* is susceptible to microevolutions during colonization and/or persistence in a specific niche, so it is possible that the type of genes and their expression depend on the site and conditions in which the bacteria colonize. This same could happen with the strains isolated in this study since different genotypes are presented; however, in general, in this study no significant differences were observed regarding the genotype and site of isolation.

An important tool for characterizing *S. aureus* strains is *spa* gene typing [66]. In this study, 62 different *spa* types were found, indicating a high genetic diversity among MRSA circulating in the studied population. This suggests that MRSA have the ability to adapt and persist in different niches of the human body. The most prevalent *spa* type was t-189, with 17 strains (6 nasal and 3 pharyngeal). It is possible that certain *spa* types may have a higher colonization capacity in the pharynx compared to the nose, which should be further studied.

The s*pa* type t-189 has been reported to be the predominant type in China [67,68] and in Ohio, USA [69], which indicates a worldwide distribution of this *spa* type. The *spa* type t-189 is associated with ST-188 strains, and strains of this type show moderate virulence with biofilm formation; however, it was found to be the dominant type in hospital bacteremia and in some foods [70], which is why this type of strain should be controlled.

The findings of this study may contribute to the development of more effective strategies for the prevention and treatment of *S. aureus* infections in different populations and colonization sites.

## 5. Conclusions

A high prevalence of *S. aureus* was found in an apparently healthy population, with greater colonization in the pharynx compared to the nose. Antibiotic resistance is significant, particularly resistance to penicillin, clindamycin, and erythromycin, and no differences were found between strains isolated from the nose and pharynx. MRSA were found with a higher prevalence of SCC*mec* type IV strains, although the percentage of CA-MRSA is low. There is a wide genotypic diversity among strains with respect to virulence factor genes and *spa* type, which apparently does not depend on the isolation site. The high prevalence of *S. aureus* pharyngeal and nasal colonization in the study population, together with the antibiotic resistance and high genetic diversity of MRSA, highlights the importance of considering both anatomical sites in studies for the prevention of *S. aureus* colonization and antibiotic resistance.

## Figures and Tables

**Table 1:** Carriers and noncarriers of *S. aureus* by age groups and anatomical sites.

Age Groups (Years) (*n* = 1777)	No Carriers (*n* = 716) (40.30%)	Both Sites (*n* = 398) (22.40%)	Exclusive Pharynx (*n* = 440) (24.75%) *^,b^	Exclusive Nose (*n* = 223) (12.55%)	Total Carriers (*n* = 1061) (59.70%)
1–10 (*n* = 499) (28.08%)	220 (44.08%)	115 (23.04%)	108 (21.64%) *^,b^	56 (11.22%)	279 (55.92%)
11–20 (*n* = 557) (31.34%)	199 (35.72%)	130 (23.33%)	155 (27.82%) *^,b^	73 (13.10%)	358 (64.28%) *^,a^
21–30 (*n* = 329) (18.51%)	117 (35.56%)	77 (23.40%)	92 (27.96%) *^,b^	43 (13.06%)	212 (64.44%) *^,a^
31–40 (*n* = 165) (9.28%)	73 (44.24%)	30 (18.18%)	35 (21.21%) *^,b^	27 (16.36%)	92 (55.76%)
41–50 (*n* = 105) (5.90%)	43 (40.95%)	24 (22.85%)	28 (26.66%) *^,b^	10 (9.52%)	62 (59.05%) *^,a^
51–60 (*n* = 49) (2.75%)	26 (53.06%)	11 (22.44%)	9 (18.36%) *^,b^	3 (6.12%)	23 (46.94%)
61–99 (*n* = 73) (4.10%)	38 (52.05%)	11 (15.06%)	13 (17.80%)	11 (15.06%)	35 (47.95%)

* *p* < 0.05; ^a^ Significant difference between carriers and non-carriers; ^b^ Significant difference between exclusive pharynx and nose carriers.

**Table 2:** MRSA isolated by site and age group.

Age Group	MRSA Pharynx	MRSA Nose	MRSA Total (*n* = 135)
1–10 (*n* = 394 strains)	18	16	34 (8.62%)
11–20 (*n* = 488 strains)	22	24	46 (9.42%)
21–30 (*n* = 289 strains)	11	17	28 (9.68%)
31–40 (*n* = 122 strains)	7	4	11 (9.01%)
41–50 (*n* = 86 strains)	4	3	7 (8.13%)
51–60 (*n* =34 strains)	2	2	4 (11.76%)
61–99 (*n* = 46 strains)	2	3	5 (10.86%)

**Table 3:** SCC*mec* typing of MRSA isolated from the pharynx and nose.

SCC*mec* Type	Pharynx Strains (*n* = 67)	Nose Strains (*n* = 68)	Total (N = 135)
II	19 (28.35%)	13 (19.11%)	32 (23.70%)
III	0	1 (1.47%)	1 (0.74%)
IV	36 (53.73%)	35 (51.47%)	71 (52.59%)
IVa	11 (16.41%)	17 (25%)	28 (20.74%)
V	1 (1.49%)	2 (2.94%)	3 (2.22%)

## Data Availability

The original contributions presented in this study are included in the article/Supplementary Materials. Further inquiries can be directed to the corresponding author.

## Supplementary Materials

The following supporting information can be downloaded at: https://www.mdpi.com/article/10.3390/microorganisms13091978/s1, Table S1: Sequences of the primers used for genotyping MRSA. Table S2: Presence of virulence factor genes in MRSA isolated from the pharynx and nose. Table S3: *spa*-typing of isolated MRSA.
