# Supplementary material for: Antibiotic Resistance and Molecular Characterization of Staphylococcus aureus Strains Colonizing the Nose and Pharynx
Source: Microorganisms. 2025 Aug 25;13(9):1978. doi: 10.3390/microorganisms13091978 (PMC12472036; doi:10.3390/microorganisms13091978)
Supplement: Supplementary file 1 [file microorganisms-13-01978-s001.zip › Supplementary Tables.pdf]

Table S1. Sequences of the primers used for genotyping MRSA strains.

Gen	Primers (5' – 3')	Amplicon (pb)	Control strain	Reference
mecA	P4: TCCAGATTACAACTTCACCAGG<br>P7: CCACTTCATATCTTGTAACG	162	ATCC 40142	[17]
*Scc*mec typing Boye et al [17]				
β<br>α3	ATTGCCTTGATAATAGCCYTCT<br>TAAAGGCATCAATGCACAAACACT	937 (II and IV)	I: BAA 44<br>II: BAA 41<br>II: BAA 39<br>IV: NRS 643<br>V: NRS 745	[18]
ccrCF<br>ccrCR	CGTCTATTACAAGATGTTAAGGATAAT<br>CCTTTATAGACTGGATTATTCAAAATAT	518 (III and V)		
1271F1<br>1272R1	GCCACTCATAACATATGGAA<br>CATCCGAGTGAAACCCAAA	415 (I and IV)		
5RmecA<br>5R431	TATACCAAACCCGACAACTAC<br>CGGCTACAGTGATAACATCC	359 (V)		
*Scc*mec typing Oliveira et al [16]				
CIF2 F2<br>CIF2 R2	TTCGAGTTGCTGATGAAGAAGG<br>ATTTACCACAAGGACTACCAGC	495 (I)	I: BAA 44<br>II: BAA 41<br>II: BAA 39<br>IV: NRS 643<br>V: NRS 745	[17]
KDP F1<br>KDPR1	AATCATCTGCCATTGGTGATGC<br>CGAATGAAGTGAAAGAAAGTGG	284 (II)		
MECI P2<br>MECI P3	ATCAAGACTTGCATTCAGGC<br>GCGGTTTCAATTCACTTGTC	209 (II and III)		
DSC F2<br>DSC R1	CATCCTATGATAGCTTGGTC<br>CTAAATCATAGCCATGACCG	342 (I, II and IV)		
RIF4 F3<br>RIF4 R9	GTGATTGTTCGAGATATGTGG<br>CGCTTTATCTGTATCTATCGC	243 (III)		
Virulence factors				
arcA	F: CACGTAACTTGCTAGAACGAG<br>R: GAGCCAGAAGTACGCGAG	724	NRS 643	[22]
lukFS-PV	F: GCATCAASTGTATTGGATAGCAAAAGC<br>R: ATCATTAGGTAAAATGTCTGGACATGATCCA	433	BAA 1556	[31]
lukED-PV	F: TGTATTCGATAGCAAAAGCAGTGCA<br>R: TGAAAAAGGTTCAAAGTTGATACGAG	269	NRS 111	[31]
luk-M	F: GAAGATATTGGCGACGATGCAGAAGT<br>R: GTATTTTGGTCTTTTGATGTAAGACC	253	ATCC 31890	[32]
psm	F: TCCTTCCTTTCGATGTCGTT<br>R: CCATCTTTTACGATGGTGGTTT	221	NRS 101	[30]
nucA	F: GATGGTGATACGGTT<br>R: AGCCAAGCCTTGACGAACTAAAGC	270	ATCC 43300	[23]
gyrA	F: AGTACATCGTCGTATACTATATGG<br>R: ATCACGTAACAGTTCAAGTGTG	283	ATCC 12600	[24]
coa	F: CACGGATACCTGTACCAGCA<br>R: ACCACAAGGTACTGAATCAACG	170	ATCC 43300	[25]
fnbA	F: CTGTGTGGTAATCAATGTC<br>R: CACAACCAGCAAATATAG	1369	ATCC 29213	[26]
fnbB	F: GCCGTCGCCTTGAGCGT<br>R: GGAGAAGGAATTAAGGCG	813	ATCC 29213	[27]
cna	F: CAGGATAGATTGGTTTA<br>R: AGTGGTTACTAATACT	560, or 1120, or 1739	NRS 123	[26]
clfA	F: CTCATCAGGTTGTTCAGG<br>R: GTAGGTACGTTAATCGGTT	1584	BAA 1556	[26]
clfB	F: TCGGTCTGTAAATAAAGGTA<br>R: TGCAAGATCAAACTGTTCCT	596	BAA 1556	[26]
icaA	F: ACTACTGCTGCGTTAATAAT<br>R: GATTATGTAATGTGCTTGGA	770	BAA 1556	[26]
icaD	F: GGCAATATGATCAAGATAC<br>R: AAACGTAAGAGAGGTGG	381	BAA 1556	[28]
srdC	F: CTGGACGGAAATATTGACCA<br>R: CGCATGGCAGTGAATACTGTTGCAGC	238	BAA 1556	[29]
sea	F: GAAAAAAGTCTGAATTGCAGGGAACA<br>R: CAAATAAATCGTAATTAACCGAAGGTTC	561	NRS 111	[31]
seb	F: ATTCTATTAAGGACACTAAGTTAGGGA<br>R: ATCCCGTTTCATAAGGCGAGT	405	NRS 266	[31]
sec	F: GTAAAGTTACAGGTGGCAAAACTTG<br>R: CATATCATACCAAAAAGTATTGCCGT	296	NRS 111	[31]
sed	F: GAATTAAGTAGTACCGCGCTAAATAATATG<br>R: GCTGTATTTTTCCTCCGAGAGT	493	NRS 110	[31]

see	F: CAAAGAAATGCTTTAAGCAATCTTAGGC<br>R: CACCTTACCGCCAAAGCTG	483	NRS 111	[31]
eta	F: ACTGTAGGAGCTAGTGCATTTGT<br>R: TGGATACTTTTGTCTATCTTTTTCATCAAC	189	NRS 266	[31]
etb	F: CAGATAAAGAGCTTTATACACACATTAC<br>R: AGTGAACTTATCTTTCTATTGAAAAACACTC	610	NRS 266	[31]
hla	F: CTGATTACTATCCAAGAAATTCGATTG<br>R: CTTTCCAGCCTACTTTTTTATCAGT	210	NRS 111	[31]
hlb	F: GTGCACTTACTGACAATAGTGC<br>R: GTTGATGAGTAGCTACCTTCAGT	310	NCTC 7428	[31]
hld	F: TTAGTGAATTTGTTCACTGTGTCGA<br>R: AAGAATTTTTATCTTAATTAAGGAAGGAGTG	111	NCTC 9393	[31]
hlg	F: GTCAYAGAGTCCATAATGCATTTAA<br>R: CACCAAATGTATAGCCTAAAGTG	533	NRS 266	[31]
tst	F: TACTAATGAATTTTTTTATCGTAAGCCCTT<br>R: TTCACTATTTGTAAAAGTGTCAGACCCACT	180	NRS 111	[31]
spa	F: TAAAGACGATCCTTCGGTGAGC<br>R: CAGCAGTAGTGCCGTTTGCTT	300 to 600	ATCC 43300	[33]

Table S2. Presence of virulence factor genes in MRSA strains isolated from the pharynx and nose

Gene	Nose strains (n= 68)	Pharynx strains (n= 67)	Total (N= 135)
	Resistance		
mecA	68 (100%)	67 (100%)	135 (100%)
	Enzymes		
arcA	15 (22.05%)	15 (22.38%)	30 (22.22%)
nucA	68 (100%)	67 (100%)	135 (100%)
gyrA	61 (89.70%)	63 (94.02%)	124 (91.85%)
	Adhesines		
fnbA	42 (61.76%)	37 (55.22%)	79 (58.51%)
fnbB	43 (63.23%)	41 (61.19%)	84 (62.22%)
cna	47 (69.11%)	49 (73.13%)	96 (71.11%)
clfA	45 (66.17%)	42 (62.68%)	87 (64.44%)
clfB	41 (60.29%)	37 (55.22%)	78 (57.77%)
coa	60 (88.23%)	62 (92.53%)	122 (90.37%)
	Biofilms		
psm	10 (14.70%)	9 (13.43%)	19 (14.07%)
icaA	65 (95.58%)	61 (91.04%)	126 (93.33%)
icaD	51 (75%)	46 (68.65%)	97 (71.85%)
sdrC	63 (92.64%)	58 (86.56%)	121 (89.62%)
	Toxins		
sea	15 (22.05%)	15 (22.38%)	30 (22.22%)
seb	6 (8.82%)	7 (10.44%)	13 (9.62%)
sec	7	11	18

	(10.29%)	(16.41%)	(13.33%)
sed	11 (16.17%)	8 (11.94%)	19 (14.07%)
see	1 (1.47%)	0	1 (0.74%)
eta	4 (5.88%)	3 (4.47%)	7 (5.18%)
etb	1 (1.47%)	1 (1.49%)	2 (1.48%)
hla	35 (51.47%)	35 (52.23%)	70 (51.85%)
hlb	18 (26.47%)	19 (28.35%)	37 (27.40%)
hld	49 (72.05%)	50 (74.62%)	99 (73.33%)
hlg	40 (58.82%)	36 (53.73%)	76 (56.29%)
tst	31 (45.58%)*	17 (25.37%)	48 (35.55%)
lukS-PV/lukF-PV	18 (26.47%)	15 (22.38%)	33 (24.44%)
***luk*E-D**	65 (95.58%)	63 (94.02%)	128 (94.81%)
***luk*-M**	0	0	0

*p<0.05

Table S3. *spa*-typing of isolated MRSA strains.

***spa*-Type**	**Nose strains (n= 68)**	**Pharynx strains (n= 67)**	**Total (n= 135)**
**t-002**	0	5	5
**t-008**	3	3	6
**t-010**	1	1	2
**t-012**	6	3	9
**t-018**	1	1	2
**t-021**	3	3	6
**t-024**	2	0	2
**t-034**	0	1	1
**t-056**	0	1	1
**t-078**	0	1	1
**t-084**	0	2	2
**t-136**	1	1	2
**t-148**	0	1	1
**t-159**	0	2	2
**t-164**	1	1	2
**t-189**	6	11	17
**t-190**	1	0	1

**t-209**	1	0	1
**t-213**	0	2	2
**t-214**	1	0	1
**t-233**	1	0	1
**t-253**	1	1	2
**t-304**	1	0	1
**t-346**	2	6	8
**t-416**	1	0	1
**t-426**	1	0	1
**t-527**	1	1	2
**t-586**	1	0	1
**t-587**	1	0	1
**t-645**	1	1	2
**t-688**	1	0	1
**t-701**	3	2	5
**t-706**	0	1	1
**t-723**	3	0	3
**t-810**	0	1	1
**t-816**	1	0	1
**t-878**	0	1	1
**t-895**	1	0	1
**t-909**	1	1	2
**t-922**	4	2	6
**t-1406**	2	2	4
**t-1710**	1	1	2
**t-1885**	1	0	1
**t-2651**	1	0	1
**t-3380**	1	0	1
**t-3955**	0	1	1
**t-4318**	1	0	1
**t-4395**	0	1	1
**t-4468**	1	1	2
**t-4656**	0	1	1
**t-4976**	2	0	2
**t-5410**	1	0	1
**t-5747**	0	1	1

**t-6367**	1	0	1
**t-8163**	1	0	1
**t-11978**	1	0	1
**t-14362**	0	1	1
**t-16143**	0	1	1
**t-16164**	0	1	1
**t-16665**	1	0	1
**t-18475**	1	0	1
